# Primiparous women’s experiences of normal vaginal delivery in Iran: a qualitative study

**DOI:** 10.1186/s12884-020-02954-4

**Published:** 2020-04-29

**Authors:** Marzieh Khamehchian, Mohsen Adib-Hajbaghery, Nastaran HeydariKhayat, Mahboubeh Rezaei, Mahdieh Sabery

**Affiliations:** 1 Trauma Nursing Research Center, Kashan University of Medical Sciences, Kashan, Iran; 2 Department of Nursing, Iranshahr University of Medical Sciences, Iranshahr, Iran; 3 Autoimmune Disease Research Center, Kashan University of Medical Sciences, Kashan, Iran

**Keywords:** Normal vaginal delivery, Qualitative study, Primiparous women

## Abstract

**Background:**

Childbirth is considered as the most challenging psychological event in a woman’s life. It has a major effect on women’s lives with long-term positive or negative impacts. Cultural, religious, and socioeconomic differences can affect women’s perception about normal vaginal delivery (NVD) experience. Therefore, it is necessary to explore the primiparous women’s perception about it.

**Methods:**

This qualitative study, with a descriptive content analysis approach, was conducted in Kashan, a city in the center of Iran. Purposive sampling was used to recruit the participants of the current study. Data was gathered by semi-structured interviews during 24 h after normal vaginal birth among primiparous women. The sampling started from June to October in 2016. Interviews continued until data saturation which was achieved in the 14th interview but for assurance, it continued until the 17th one.

**Results:**

The following three main themes were extracted “immersion in stress”, “pain, the essence of NVD” and “strategies for situation management”. Furthermore, seven subthemes were obtained including ‘loss threat’, ‘stressful context’, temporary impairment in physiologic harmony, paradoxical emotions, self-management, emotional support, and spiritual support.

**Conclusions:**

This study showed that stress and pain were two highlighted issues in NVD process. Increasing women’s awareness about NVD process, familiarizing the primiparous women with the simulated delivery room, accompanying these women for emotional support, and providing spiritual support can be effective in situation management to make the child delivery a pleasant and satisfying experience.

## Background

Birth is the most challenging physiological and psychological events in the women’s life [1, 2]. Childbirth is not only a transition to motherhood, but also it is associated with considerable physical and emotional impacts in a mother’s life [3]. It has a powerful effect on women’s lives with long-term positive or negative effects [4, 5]. A positive birth experience can have long-lasting profits such as the improvement of the relationship between a mother and a child, the development of parents’ well-being, self-confidence, and the quality of life [2, 6]. In contrast, a negative birth experience could affect their sense of motherhood and womanhood [7]. It is associated with negative health outcomes, post-traumatic stress disorder, decrease in rates of exclusive breastfeeding, interruption to social relationships, dysfunction in mother-infant relationships, fear of childbirth, increased tendency for an elective caesarean section in future pregnancies [1, 2, 5, 7].

The first child’s birth and the choice of delivery type are considered as the most important events for the primiparous women. The normal vaginal delivery (NVD) as a normal physiological process has many benefits for both the mother and the child [8]. Today, cesarean section is the first option for primiparous women in most countries especially in Iran [9]. Cesarean section, in comparison with NVD, has many dangerous consequences for the mother and the child [8, 10].

Based on the evidence, religious, cultural, and socioeconomic factors influence the perception of delivery and childbirth [11]. Aziato et al. (2016) also considered that religious and traditional beliefs cannot be separated from the childbirth [12]. As majority of Iranians are Muslim (98%), religion and spirituality play an important role in all aspects of life, especially in pregnancy and childbirth [13]. During pregnancy, women intensify their prayers to assure their God’s protection, safe delivery and blessings. It affords them the confidence of going through a safe delivery [12]. Muslim women believe that their sins would be forgiven because of the pain and the hardship of NVD. Also, they state that God gives Heaven to women who have a normal child birth.

In recent years, the rate of cesarean section is 48% in Iran that has increased to 87% in some private hospital [8, 14], most of which are without medical indication [8, 15]. The causes for the high rate of caesarian section have been studied [8, 16–20]. Sahlin et al. (2013) mentioned that negative child birth experience is one of the contributing factors on women’s tendency for choosing cesarean section [21]. Evidence showed that 10–20% of all women have negative birth experiences [7].

The reason why the rate of cesarean delivery has increased so dramatically in Iran is not entirely clear. It is important to know what has happened over the recent years. What has changed that despite the religious context in Iran, the tendency of primiparous women with no prior birth experience is less likely to NVD? Exploring women’s perception about normal childbirth experiences can enable policy-makers to identify the factors removing undesirable and negative experiences of childbirth and thus facilitate a pleasant childbirth experience for mothers through educational and structural interventions. In addition, through the recognition of women’s perception of NVD, societies can be directed towards a positive understanding of vaginal delivery, which can ultimately lead to maternal health promotion. Pazandeh et al. (2017) stated that improving the quality of care and creating a positive experience of NVD with minimal complications should be the primary goal of health care providers [22]. Therefore, considering the cultural, religious, and socioeconomic differences in the Iranian context, it seems necessary to explore the primiparous women’s perception toward NVD experience.

## Methods

A descriptive qualitative design using a content analysis approach was conducted to extract the experience of Iranian women’s experience regarding normal vaginal delivery. Data gathering lasted from June to October 2016. The context of data gathering was a state hospital in Kashan, a city in the center of Iran.

### Ethical considerations

The Institutional Review Board and the Research Ethics Committee of Kashan University of Medical Sciences (Project No. 93230) approved the study protocol. Permissions were also obtained from the hospital data authorities.

The participants were informed about the aims, the importance of the study and withdrawal from the study at any time. They were also assured about the confidentiality of the provided data. The names of all participants were changed into codes while transcribing the interviews and the data related to each interview was kept in a safe place. All participants signed an informed written consent before taking part in the study.

### Participants

The participants were selected with a purposive sampling among primiparous women in postpartum ward. The present study was conducted 24 h after vaginal birth and before discharging from the hospital (because at this time the experience of natural childbirth is fresh and memorable). Data gathering lasted from June to October 2016. Inclusion criteria were 19- to 34-year-old primiparous women with no history of psychological disorders, gestational age of 37–40 weeks, cephalic presentation, and no history of abortion, and ectopic pregnancy. However, the use of drug for induction, and the deterioration of mother’s status and the need for extra care constituted the exclusion criteria. The primiparous women with no labor experience were selected for this study because based on evidence, the previous experience of labor in the multipara women can affect perception, feelings, and the choice of the delivery type [23].

### Data collection

Face-to-face semi-structured interviews with open-ended questions were conducted to explore the detailed perception of primiparous women. The main questions of the interview guide included “Would you please explain about your experiences of NVD?”, “How did you perceive NVD?”, “How did you feel when you were in the delivery room?” and “Can you talk about your experiences when the baby was delivering?”

Then, the researchers explored more about the participants’ perceptions by using probing questions such as “Please explain more about it” or “Could you please give us an example?”, and “What do you mean by this?”

The interviews were conducted by the first author in one or more sessions. An interview guide was developed in this study, shown in the supplementary file 1. All interviews were conducted in Persian in a quiet and private room in post-partum ward. The length of each interview lasted from 30 to 50 min. Interviews continued till data saturation i.e. repetitive data with no extraction of new conceptual codes. Data saturation was achieved in the 14th interview based on the consensus between all researchers. However, for more assurance, the interviews continued until the 17th one but no new codes were obtained.

### Data analysis

Qualitative content analysis study was used to achieve a rich description of the phenomenon of interest in a natural context **(**22**)**. Data analysis was conducted concurrently with data collection, so each interview was followed by data analysis in the same day. In order to do so, the interviews were transcribed verbatim and read several times to reach an overall understanding of women’s perspectives on normal vaginal birth. Meaning units, including words, sentences or paragraphs relevant to women’s perceptions of NVD were extracted and labeled with codes. Then, the codes were sorted into clusters of themes and sub-themes based on their similarities and differences. Finally, themes or expressions of latent content were obtained **(**24**)**. An example of analysis phases is shown in Table 1.

**Table 1:** An example of interview analysis phases using content analysis approach

Significant statements	Meanings	Sub-theme	theme
“I knew that I had to bear the pain to reach the desired outcome”	Acceptance of self-management of labor pain	Self-management	Strategies for management situation

### Trustworthiness

The credibility of the data was established through peer check and member check. Peer checking was conducted by two expert supervisors from Kashan University of Medical Sciences to verify the coding and categorization process. For member checking, some interview drafts were returned to the participants to verify the correct perceptions. In order to promote the transferability, the researchers focused on clarification, reflexivity, and neutrality. Furthermore, the demographic characteristics of the study population were described in detail. The researchers attempted to increase the conformability through keeping all the documentations at all stages and providing exact reports in order to generalize the results for further studies [24].

## Results

Totally, 17 primiparous women took part in the study. The age of the participants ranged from 19 to 34 years old. Furthermore, the majority of participants were living in an urban area with average income, different educational level, and medical insurance. As illustrated in Table 2, three main themes were emerged from data including “Immersion in stress”, “Pain, the essence of NVD”, and “Strategies for situation management” which indicated the nature and dimensions of the women’ perceptions of NVD.

**Table 2:** Summary of themes and clusters of themes

Themes	Sub-themes	Clusters of sub-themes
**Immersion in stress**	Loss threat	Fear of damage to the newborn
		Fear of abnormal newborn
		Fear of death
		Fear of damage to genital organs
	Stressful context	Concern induced by unfamiliar environment
		Induced concerns
**Pain, the essence of NVD**	Temporary impairment in physiologic harmony	
	Paradoxical emotions	Pleasant experiences
		Unpleasant experiences
**Strategies for situation management**	Self-management	
	Emotional support	
	Spiritual support	

### “Immersion in stress”

“Immersion in stress” is the most important theme identified from the participants’ responses. This theme consists of two sub-themes including ‘loss threat’, and ‘stressful context’.

#### ‘Loss threat ‘

The category of ‘Loss threat’ was the most common experience of NVD among the primiparous women. This sub-theme comprised four clusters of sub-themes including ‘fear of damage to the infant during childbirth’, ‘fear of abnormal infant’, ‘fear of death’, ‘fear of damage to genital organs’.

The participants declared that most of the concerns were due to words of others and past experiences.

As one participant said: *“My neighbor’s daughter had a NVD last year and because of the baby’s head injury, his brain was damaged and he would be paralyzed for the rest of his life. I’m afraid of having giving birth because of the risk of injury to baby” (participant 3).* Another participant stated: *“My sister had difficulty in sitting because of excessive rupture in the perineum during NVD”(participant 2).*

#### Stressful context

Many of the women’s concerns were related to being in a strange and unfamiliar environment. The participants described the delivery environment full of noises, annoying sounds, unpleasant feelings, stress, pain and loneliness and an environment without intimacy. One of the participants mentioned: *“I was very afraid of the delivery room. I had never experienced such an environment before. Nothing was familiar to me. I was afraid of staff and special equipment in the delivery room; I was even afraid of the smells and green colors of the walls”(participant 8).* Another mother added: *“I was horrified by the screams of the women who were giving birth. These screams made me more stressed. I would have preferred an environment with no disturbing noises”(participant 11).*

The second effective element which was related to “stressful context” was “concerns induced by the words of others”. It was due to being influenced by others’ comments and experience. Indeed, it was an imagination created by others. It seems that not only did the maternal experience of NVD not create a pleasant/positive picture for primiparous women, but it also presented a terrifying image about normal childbirth.

Occasionally, these concerns induced by the words of others were so intense that resulted in the imposed unrealistic inability to do NVD in women. Also, the presence of other women who were shouting due to labor pain in delivery room was a source of induced concern for primiparous mothers.

A major part of concerns was inevitable in normal delivery process. These concerns might alter women’ self confidence and self-esteem, and even might destroy them. One of the participants said: *“Two of my grandmother’s babies died during normal delivery. My mom and my aunt had a difficult NVD, all of which worried me”(participant 9).*

### Pain, the essence of NVD

This theme showed that pain is an integral and inseparable component of NVD. It means that NVD without pain is impossible. One participant stated it: *“Everyone who wants to talk about NVD starts straight with its pain.”(Participant 2). The pain of normal labor is different from any other pains. It seems that you cannot imagine a normal delivery without pain. “Sometimes we hear about painless labor, but I think it is just a slogan. I cannot believe that there is any normal delivery without pain”.***(***Participant 4).*

This theme included two sub-themes: “temporary impairment in physiologic harmony” and “paradoxical emotions”.

#### Temporary impairment in physiologic harmony

The majority of participants experienced vomiting, shivering, dyspnea, amnesia, fatigue, thirst, lack of concentration and loss of control. It is supposed that the physiological harmony of the body is temporarily impaired during the NVD process. As one of the participants added, “*I felt short of breath and they gave me oxygen. However, it did not help me and I had a sense of choking”. “My pain was so severe and persistent that I lost concentration”. (Participant 1).*

Moreover, some women felt these painful and difficult conditions as awful as feeling of imminent death. One participant said: *“The pain of NVD was so horrible that I felt I was going to die soon”.****(****Participant 3).*

#### Paradoxical emotions

This sub-theme was divided into two clusters of subthemes: pleasant experiences and unpleasant experiences. Most participants explained a combination of pleasant and unpleasant experiences simultaneously which created paradoxical emotions. Some of participants described that the unpleasant experiences of NVD were accompanied by a sweet feeling of motherhood, the feeling of having a healthy normal infant, and the feeling of success and accomplishment. One of the participants said: *“This process was painful, but it had a sweet ending*”. (Participant 2). Another participant stated: *“This made me able to tolerate the horrible pain when I thought my baby was going to be born at the end of labor pain*”. (Participant 5). Another woman said: *“I experienced all the emotions that you can’t imagine, once I was* screaming, *groaning, moaning and crying and another time I was laughing. It was a very difficult time for me but as soon as I saw the baby, I felt it was worth it. That’s the most beautiful and wonderful moment.”* (Participant 15).

### Strategies for situation management

Women during NVD process develop and adopt various strategies to manage this situation. This theme contained three sub-themes: ‘self-management’, ‘emotional support’ and ‘spiritual support’.

#### Self-management

The participants believed that self-management in NVD is a useful factor for situation management. They considered themselves as responsible people for achieving the desired success in NVD process. One of the participants quoted one of the midwives who had said *“you are mainly responsible for the birth of your baby. We are merely facilitators” (Participant 13).* Another participant said: *“A midwife told me, if you want to have an easy and comfortable child birth, you must be calm and cooperate with me”. She also said: “do not shout and just take a deep breath to save your energy”. I did what she said, but in the delivery room, there was a woman who did not listen to her midwife and she failed in NVD. Eventually, she was transferred to the operation room for cesarean* section (Participant 3).

#### Emotional support

The participants said that the need for emotional support was not limited to health care providers (HCPs); if their spouse, mother, and sister had attended there, they would have felt more comfortable and satisfied. They also added the presence of their family members facilitated the pain relief, the tolerance to difficult condition and eventually caused the feeling of pleasant experience. One participant stated: *“The presence of a person like mother, sister, spouse, or a friend is very effective and peaceful)” participant 6).*

#### Spiritual support

Spiritual support is the third sub-theme of “strategies for situation management”. Spiritual support originates from religious beliefs of participants, especially from their faith in God and holy people.

One of the participants mentioned: “*when I had pain, I prayed and I felt I gained more power to tolerate the severe pain*” (Participant 2). Another participant added “*during labor pain, I called God and holy people repeatedly, I felt less pain and I could handle the pain more easily*” (Participant14). Most participants believed that faith in God promotes self-esteem and self-actualization. Some of them even believed that labor pain is an opportunity for spiritual growth and a way to connect to God. One of the participants asserted: *“Muslims believe that the sins of women will be forgiven during labor pain”*. (Participant 6).

## Discussion

This qualitative study aimed to explore the primiparous women’s experiences of NVD process. “Immersion in stress” was considered as the main theme of the present study. “Immersion in stress” was associated with two factors including loss threat and stressful context. ‘Fear of damage to the infant during childbirth’, ‘fear of abnormal infant’, ‘fear of death’, ‘fear of damage to genital organs’ were the sources of stress in the present study. In line with the previous studies, our findings indicated that the women in labor were excessively concerned about their infants and maternal complications during the normal childbirth [9, 25–28]. Ravangard et al. (2017) stated that normal birth is inevitably influenced by emotional, social, and psychological stresses that inhibit pleasant feeling during NVD process [29]. McLeish & Redshaw (2019) also concluded that psychosocial stress of normal childbirth can cause poor emotional wellbeing and hinder successful adaptability to the maternal role [30]. Elvander et al. (2013) believed women with high fear of childbirth have reported the lowest pleasant experience [31].

What is inferred from the participants’ statements is that they described the delivery environment as an unfamiliar and noisy place, an environment full of pain, fear, anxiety, and without intimacy and friendship. Screaming and shouting of other women who are giving birth in this environment worsen the fear and the anxiety. Khatony et al. (2019) argued that one of the reasons of fear during delivery is related to the hospital environment and low quality of care [9]. Mahmood (2016) stated that unfamiliar, crowded and noisy environment of hospitals make childbirth an unpleasant experience [32]. Akadri and Odelola, (2018) stated that environmental factors and the degree of strangeness of the environment (equipment, sound, light and restrictiveness) have a great effect on labor pain [33]. Based on evidence, interactions among physiologic, psychosocial and environmental factors affect women’s perceptions of labor pain [32]. Women in labor are highly vulnerable. They are in a strange environment, their private body parts are exposed; they have an awful pain and they hear the sighing and screaming of other women in delivery room. They are alone and no family members are present. Based on “fear–tension–pain” theory which was theorized by Grantley Dick-Read, the pain and anxiety during labor leads to the release of catecholamine, which reduce uterine contractions, increase risk of prolonged labor and fetal distress [32, 34]. Thus, in order to create a pleasant perception of NVD, we need to implement procedures to reduce stress, establish a friendly environment and allow the presence of a family member, a friend or a relative.

The result showed that hearing bitter experiences of other women regarding NVD process was the main source of concern in the primiparous women in this study. Latifnejad Roudsari et al. (2015) argued that these birth stories are mostly associated with unpleasant aspects of childbirth, such as psychological anxiety, fear, severe pain, and inappropriate interventions [35]. These negative aspects are also highlighted by social media. Azami-aghdash et al. (2014) showed that social factors are the main determinant of women’s unwillingness to give a normal birth [16]. Review of liturature in Iranian context demonstrated that sociocultural factors have important roles in the selection of delivery type [13, 28, 35]. Based on the available evidence, social factors and incorrect methods of information broadcasting are the most important factors affecting the choice of delivery mode among Iranian primiparous women [9].

Another important experience associated with NVD process was severe labor pain which converted the pleasant feeling of delivery into unpleasant experiences. Women described labor pain as the most horrible pain that they had ever experienced. Majority of studies also describe labor pain as the most severe pain experienced by women [8, 9, 36]. Labor pain is an important concern for every pregnant woman and the inability to relieve it might have a a significant impact on birth outcomes [37]. Based on women’s experiences, labor pain is an inseparable and inevitable component of NVD process. It has been reported that 40% of multiparous women and 60% of primiparous women had experienced severe pain in labor phase [37]. Evidence showed that fear of labor pain is one of the main reasons for selecting cesarean delivery by women [16, 36, 37].

In addition, paradoxical emotions along with pain were experienced by the participants during NVD process. They described labor pain so severe that disturbed the physiological harmony of their body. Boryri et al. (2016) stated that labor pain decreases women’s energy, power and control of the situation [4]. What separates this pain from other pains is that labor pain is followed by a sweet feeling of being a mother**.** As the pain deteriorates, the mother becomes closer to the baby. In other words, this unpleasant feeling of pain is associated with a sweet ending to having a baby. Callister et al. (2003) maintained that the labor pain differs from the acute or chronic pain of a disease, trauma, and surgical procedures. This pain is worthwhile for women because it bestowed the gift of motherhood to them [38].

The current study showed that even though the pain is the essence of NVD, the women attempted to use various strategies to manage the situation. Thomson et al. argued that owing to multi-dimensional nature of labor pain, women with different socio-cultural backgrounds have to use various coping strategies to adapt with this condition [30]. Self-management, emotional support and spiritual support were important approaches that assisted the participants to cope with this particular situation. Larkin et al. (2012) considered that self-management is a key component of the NVD process and a major predictor of the sense of satisfaction and success among women [39]. Wilson and Simpson (2016) also indicated that women with the ability to cope with psychological anxieties felt a less labor pain [40]. The women in Downe et al’s study considered that NVD can be a fearful and unpredictable event, but women have to maintain personal control to manage the situation [41].

Emotional support is another effective approach for situation management. Kozhimannil et al. (2013) concluded that the emotional support provided by “Doula” can relieve the labor pain, increase the satisfaction, and create a pleasant experience and positive outcome [42, 43]. Doula is an experienced woman who provides continuous emotional support to women during childbirth [30, 43–45]. Moreover, Boryri et al. (2016) stated that ongoing support from Doula or one of the family members reduced the medical interventions, increased the mother’s satisfaction, enhanced the neonatal outcomes, and improved the psychological wellbeing of mothers [4]. In Iran, it is not possible to attend a baby’s fathers in labor. If these conditions are met, it will be very effective in creating a pleasant experience and reducing fear and anxiety of primiparous women.

Spiritual support was considered as the main approach for situation management among Iranian women. It seems that this concept is originated from religious context in Iran because as mentioned before the majority of Iranian women are Muslim. Based on the evidence, Faith in God is a connection to the superior power that enhances the feeling of control and intrinsically gives power to women [12]. Mutmainnah and Afiyanti (2019) in their study about Muslim women’s experience of spirituality during pregnancy concluded that faith in God enhances self-confidence, self-control, and persistence during delivery and women can overcome the challenge of childbirth [46]. Tork Zahrani et al. (2019) considered spiritual health as a valuable coping strategy for adapting to pregnancy and childbirth [13]. Akadri and Odelola (2018) suggested that religious beliefs may empower women to tolerate the labor pain [33]. Mohamadirizi et al. (2018) argued that religious-spiritual support can increase self-efficacy among women in child birth [47]. Therefore, the planning and establishing an effective training course about religious-spiritual support are recommended.

### Limitations

This study used a descriptive content analysis approach that employed individual in-depth interviews to explore the perception of primiparous women of NVD process. Although the qualitative approach generated a deep insight into the phenomenon under study, the recruitment of only 17 women from an urban community who were Muslim made the generalization of findings to all primiparous women in Iran difficult. It is assumed that women living in different religious context would have different experiences about vaginal birth process.

## Conclusions

This study showed that stress and pain were two highlighted issues in NVD process. Increasing women’s awareness of NVD process and what they experience during this process, familiarizing the primiparous women with the simulated delivery room, accompanying these women for emotional support, and providing spiritual support can be effective in situation management to make the child delivery a pleasant and satisfying experience.

## Supplementary information

**Additional file 1.**

## Data Availability

The Ethical Review Board approval was obtained for public sharing and presentation of data on group level only. This means that the data used in this study can only be used for the approved research and cannot be shared by the authors.

## Abbreviation

NVD: Normal Vaginal Delivery

## References

[1] Taheri M, Takian A, Taghizadeh Z, Jafari N, Sarafraz N (2018). Creating a positive perception of childbirth experience: systematic review and meta-analysis of prenatal and intrapartum interventions. Reprod Health.

[2] Guittier M-J, Cedraschi C, Jamei N, Boulvain M, Guillemin F (2014). Impact of mode of delivery on the birth experience in first-time mothers: a qualitative study. BMC Pregnancy Childbirth.

[3] Bertucci V, Boffo M, Mannarini S, Serena A, Saccardi C, Cosmi E (2012). Assessing the perception of the childbirth experience in Italian women: A contribution to the adaptation of the childbirth perception questionnaire. Midwifery.

[4] Boryri T, Noori NM, Teimouri A, Yaghobinia F (2016). The perception of primiparous mothers of comfortable resources in labor pain (a qualitative study). Iran J Nurs Midwifery Res.

[5] Nilvér H, Begley C, Berg M (2017). Measuring women’s childbirth experiences: a systematic review for identification and analysis of validated instruments. BMC Pregnancy Childbirth.

[6] Hildingsson I, Johansson M, Karlström A, Fenwick J (2013). Factors associated with a positive birth experience: an exploration of swedish women’s experiences. Int J Childbirth.

[7] Smarandache A, Kim TH, Bohr Y, Tamim H (2016). Predictors of a negative labour and birth experience based on a national survey of Canadian women. BMC Pregnancy Childbirth.

[8] Rafiei M, Ghare MS, Akbari M, Kiani F, Sayehmiri F, Sayehmiri K (2018). Prevalence, causes, and complications of cesarean delivery in Iran: a systematic review and meta-analysis. Int J Reprod Biomed.

[9] Khatony A, Soroush A, Andayeshgar B, Saedpanah N, Abdi A (2019). Attitude of primiparous women towards their preference for delivery method: a qualitative content analysis. Arch Public Health.

[10] Akbari S, Ahmadi S (2014). Analyzing the effective factors of choosing delivery method of the primiparous pregnant women attending to Khoramabad’s Asalian hospital in 2014. Indian J Fundamental Appl Life Sci.

[11] Shahoei R, Rezaei M, Ranaei F, Khosravy F, Zaheri F (2014). K urdish women's preference for mode of birth: a qualitative study. Int J Nurs Pract.

[12] Aziato L, Odai PN, Omenyo CN (2016). Religious beliefs and practices in pregnancy and labour: an inductive qualitative study among post-partum women in Ghana. BMC Pregnancy Childbirth.

[13] Zahrani ST, Rafiei EH, Hajian S, Majd HA, Izadi A (2020). The Correlation between Spiritual Health and Maternal-Fetal Attachment Behaviors in Pregnant Women Referring to the Health Centers in Qazvin, Iran.

[14] Jafarzadeh A, Hadavi M, Hasanshahi G, Rezaeian M, Vazirinejad R, Aminzadeh F (2019). Cesarean or Cesarean Epidemic?. Arch Iran Med.

[15] Betrán AP, Temmerman M, Kingdon C, Mohiddin A, Opiyo N, Torloni MR (2018). Interventions to reduce unnecessary caesarean sections in healthy women and babies. Lancet.

[16] Azami-Aghdash S, Ghojazadeh M, Dehdilani N, Mohammadi M (2014). Prevalence and causes of cesarean section in Iran: systematic review and meta-analysis. Iran J Public Health.

[17] Faisal I, Matinnia N, Hejar A, Khodakarami Z (2014). Why do primigravidae request caesarean section in a normal pregnancy? A qualitative study in Iran. Midwifery..

[18] Khayyatian N, Nasiri S (2016). Prevalence of cesarean section and its causes in governmental obstetric hospitals of Kashan-2014.

[19] Ghanbari Z, Shokouhmand R, Asadi H, Naghizadeh S, Chupani F (2014). Assessing the relative contribution of causes leading to cesarean section among women who had referred to Shahid Madani Clinic in Marand city.

[20] Sotoodeh Jahromi A, Rahmanian K, Madani A (2018). Relation of knowledge about cesarean disadvantages and delivery mode selection in women with first pregnancy; south of Iran. J Res Med Dent Sci.

[21] Sahlin M, Carlander-Klint A-K, Hildingsson I, Wiklund I (2013). First-time mothers' wish for a planned caesarean section: deeply rooted emotions. Midwifery..

[22] Pazandeh F, Potrata B, Huss R, Hirst J, House A (2017). Women’s experiences of routine care during labour and childbirth and the influence of medicalisation: a qualitative study from Iran. Midwifery..

[23] Pirdil M, Pirdel L (2015). A comparison of women’s expectations of labour and birth with the experiences in primiparas and multiparas with normal vaginal delivery. J Kathmandu Med College.

[24] Graneheim UH, Lundman B (2004). Qualitative content analysis in nursing research: concepts, procedures and measures to achieve trustworthiness. Nurse Educ Today.

[25] Raksha G, Anjali T, Kirna T (2017). An exploratory study to assess the factors causing anxiety among Primigravida planned for Normal vaginal delivery and caesarean section admitted at Mata Kaushalya hospital, Patiala, Punjab. Maternal Pediatr Nutrition.

[26] Misaeli C, Kamala B, Mgaya A, Kidanto HL (2017). Factors associated with women’s intention to request caesarean delivery in Dar Es Salaam, Tanzania. S Afr J Obstet Gynaecol.

[27] Ghotbi F, Akbari Sene A, Azargashb E, Shiva F, Mohtadi M, Zadehmodares S (2014). Women's knowledge and attitude towards mode of delivery and frequency of cesarean section on mother's request in six public and private hospitals in T ehran, I ran, 2012. J Obstet Gynaecol Res.

[28] Zakerhamidi M, Shoarinejad S, Mohammadpour S (2014). Fe3O4 nanoparticle effect on dielectric and ordering behavior of nematic liquid crystal host. J Mol Liq.

[29] Ravangard R, Basiri A, Sajjadnia Z, Shokrpour N (2017). Comparison of the effects of using physiological methods and accompanying a doula in deliveries on nulliparous women's anxiety and pain: a case study in Iran. Health Care Manag.

[30] McLeish J, Redshaw M (2019). Maternity experiences of mothers with multiple disadvantages in England: a qualitative study. Women Birth.

[31] Elvander C, Cnattingius S, Kjerulff KH (2013). Birth experience in women with low, intermediate or high levels of fear: findings from the first baby study. Birth..

[32] Mahmood K (2016). Labour and birth experiences and awareness of pain relief among Kurdish women: University of Sheffield.

[33] Akadri AA, Odelola OI. Labour pain perception: experiences of Nigerian mothers. Pan Afr Med J. 2018;30(1). 10.11604/pamj.2018.30.288.16672 PMC6320448 30637072

[34] Safarzadeh A, Beigi M, Salehian T, Khojasteh F, Burayri T, Navabirigi S (2012). Effect of doula support on labour pain and outcomes in Primiparous women in Zahedan, southeastern Iran: a randomized controlled trial. J Pain Relief.

[35] Roudsari RL, Zakerihamidi M, Khoei EM (2015). Socio-cultural beliefs, values and traditions regarding women’s preferred mode of birth in the north of Iran. Int J Commun Based Nurs Midwifery.

[36] Zamani-Alavijeh F, Araban M, Hassanzadeh A, Makhouli K (2018). Contributing factors of pregnant women’s beliefs towards mode of delivery: a cross-sectional study from Iran. Maternal Health Neonatol Perinatol.

[37] Taghinejad H, Delpisheh A, Suhrabi Z (2010). Comparison between massage and music therapies to relieve the severity of labor pain. Womens Health.

[38] Callister LC, Khalaf I, Semenic S, Kartchner R, Vehvilainen-Julkunen K (2003). The pain of childbirth: perceptions of culturally diverse women. Pain Manag Nurs.

[39] Larkin P, Begley CM, Devane D (2012). ‘Not enough people to look after you’: an exploration of women's experiences of childbirth in the Republic of Ireland. Midwifery..

[40] Wilson CL, Simpson JA (2016). Childbirth pain, attachment orientations, and romantic partner support during labor and delivery. Pers Relat.

[41] Downe S, Finlayson K, Oladapo O, Bonet M, Gülmezoglu AM (2018). What matters to women during childbirth: a systematic qualitative review. PLoS One.

[42] Hodnett ED, Gates S, Hofmeyr GJ, Sakala C. Continuous support for women during childbirth. Cochrane Database Syst Rev. 2013;7. 10.1002/14651858.CD003766.pub5 23857334

[43] Butler MM (2017). Exploring the strategies that midwives in British Columbia use to promote normal birth. BMC Pregnancy Childbirth.

[44] Hans SL, Thullen M, Henson LG, Lee H, Edwards RC, Bernstein VJ (2013). Promoting positive mother–infant relationships: a randomized trial of community doula support for young mothers. Infant Ment Health J.

[45] Stevens J, Dahlen H, Peters K, Jackson D (2011). Midwives’ and doulas’ perspectives of the role of the doula in Australia: a qualitative study. Midwifery..

[46] Mutmainnah M, Afiyanti Y (2019). The experiences of spirituality during pregnancy and child birth in Indonesian muslim women. Enferm Clin.

[47] Mohamadirizi S, Mohamadirizi M, Mohamadirizi S, Mahmoodi F. The effect of religious-spiritual support on childbirth self-efficacy. J Educ Health Promot. 2018;7. 10.4103/jehp.jehp_60_17 PMC5791441 29417074

