# Supplementary material for: Primiparous women’s experiences of normal vaginal delivery in Iran: a qualitative study
Source: BMC Pregnancy Childbirth. 2020 Apr 29;20:259. doi: 10.1186/s12884-020-02954-4 (PMC7191821; doi:10.1186/s12884-020-02954-4)
Supplement: Supplementary file 1 — Additional file 1. [file 12884_2020_2954_MOESM1_ESM.docx]

**Interview guide**

- Main question: "Would you please explain about your experiences of NVD?"
- "How did you perceive NVD?"
- "How did you feel when you were in the delivery room?”
- “Can you talk about your experiences when the baby was delivering?"
- Probing questions: “Please explain more about it”
- “Could you please give us an example?”
- “What do you mean by this?”
